# Mendelian randomization provides support for obesity as a risk factor for meningioma

**DOI:** 10.1038/s41598-018-36186-6

**Published:** 2019-01-22

**Authors:** Hannah Takahashi, Alex J. Cornish, Amit Sud, Philip J. Law, Linden Disney-Hogg, Lisa Calvocoressi, Lingeng Lu, Helen M. Hansen, Ivan Smirnov, Kyle M. Walsh, Johannes Schramm, Per Hoffmann, Markus M. Nöthen, Karl-Heinz Jöckel, Joellen M. Schildkraut, Matthias Simon, Melissa Bondy, Margaret Wrensch, Joseph L. Wiemels, Elizabeth B. Claus, Clare Turnbull, Richard S. Houlston

**Affiliations:** 1 Division of Genetics and Epidemiology, The Institute of Cancer Research, London, UK; 2 School of Public Health, Yale University, New Haven, Connecticut USA; 3 Division of Neuroepidemiology, Department of Neurological Surgery, University of California San Francisco, San Francisco, California USA; 4 University of Bonn Medical School, Bonn, Germany; 5 Human Genomics Research Group, Department of Biomedicine, University of Basel, Basel, Switzerland; 6 Department of Genomics, Life & Brain Center, University of Bonn, Bonn, Germany; 7 Institute of Human Genetics, University of Bonn School of Medicine & University Hospital Bonn, Bonn, Germany; 8 Institute for Medical Informatics, Biometry and Epidemiology, University Hospital Essen, University of Duisburg-Essen, Essen, Germany; 9 Department of Public Health Sciences, University of Virginia, Charlottesville, Virginia USA; 10 Department of Neurosurgery, Bethel Clinic, Bielefeld, Germany; 11 Section of Epidemiology and Population Sciences, Department of Medicine and Dan L., Duncan Comprehensive Cancer Center, Baylor College of Medicine, Houston, Texas USA; 12 Institute for Human Genetics, University of California San Francisco, San Francisco, California USA; 13 Department of Epidemiology and Biostatistics, University of California San Francisco, San Francisco, California USA; 14 Department of Neurosurgery, Brigham and Women’s Hospital, Boston, Massachusetts USA; 15 William Harvey Research Institute, Queen Mary University, London, UK; 16 Guys and St. Thomas Foundation NHS Trust, Great Maze Pond, London, UK

## Abstract

Little is known about the causes of meningioma. Obesity and obesity-related traits have been reported in several epidemiological observational studies to be risk factors for meningioma. We performed an analysis of genetic variants associated with obesity-related traits to assess the relationship with meningioma risk using Mendelian randomization (MR), an approach unaffected by biases from temporal variability and reverse causation that might have affected earlier investigations. We considered 11 obesity-related traits, identified genetic instruments for these factors, and assessed their association with meningioma risk using data from a genome-wide association study comprising 1,606 meningioma patients and 9,823 controls. To evaluate the causal relationship between the obesity-related traits and meningioma risk, we consider the estimated odds ratio (OR) of meningioma for each genetic instrument. We identified positive associations between body mass index (odds ratio [OR_SD_] = 1.27, 95% confidence interval [CI] = 1.03–1.56, *P* = 0.028) and body fat percentage (OR_SD_ = 1.28, 95% CI = 1.01–1.63, *P* = 0.042) with meningioma risk, albeit non-significant after correction for multiple testing. Associations for basal metabolic rate, diastolic blood pressure, fasting glucose, high-density lipoprotein cholesterol, low-density lipoprotein cholesterol, systolic blood pressure, total cholesterol, triglycerides and waist circumference with risk of meningioma were non-significant. Our analysis provides additional support for obesity being associated with an increased risk of meningioma.

## Introduction

Meningioma is the commonest form of brain tumour, affecting approximately one per cent of the adult population^1^. Although mortality from meningioma is generally low, the disease is associated with substantial morbidity.

Thus far, little is known about the aetiological basis of meningioma with few well-established risk factors for the disease^2,3^. There is increasing recognition that obesity and obesity-related traits, such as body mass index (BMI), are risk factors for common cancers such as breast, colon, ovarian, renal and pancreatic^4^. Observational epidemiological studies have however provided inconsistent evidence for obesity and obesity-related traits being risk factors for meningioma^5–12^. Moreover, in contrast to many cancers, some studies have reported an inverse relationship between hyperglycaemia, type-2 diabetes and meningioma risk^13–15^. Such observational epidemiological studies can however be influenced by reverse causation, biasing findings.

Mendelian randomisation (MR) uses genetic variants as instrumental variables (IVs) to assess the causal relationship between an exposure, such as a lifestyle-related or environmental risk factor, and an outcome, such as the risk of a disease^16^. These genetic variants represent unconfounded markers of exposure, provided that these instruments are not associated with disease risk through an alternative mechanism^16^. As these genetic variants are assigned randomly at conception, they are not influenced by reverse causation, and MR therefore represents an approach orthogonal to traditional observational epidemiological studies. If the assumptions of MR are not violated, then the association between the genetic instruments and the outcome of interest indicates a causal relationship between the exposure and outcome. Parallels have been drawn between MR and randomised controlled trails, in that the random assignment of variants at conception similarly circumvents some of the limitations of observational epidemiological studies^17^.

Many genetic instruments explain only a small proportion of exposure variation^18^ and MR frameworks have therefore been developed that consider multiple genetic variants when estimating the causal effect of an exposure on an outcome^19^. Each additional independent single nucleotide polymorphism (SNP) considered in a multi-SNP approach provides further information on the causal relationship, and combining all valid genetic instruments thereby affords a more precise estimation of the causal effect^19^.

To further our understanding of meningioma aetiology, we implemented a Generalised Summary-data-based Mendelian Randomisation (GSMR) to evaluate the causal relationship between 11 key obesity-related traits and meningioma risk^20^. This multi-SNP MR approach is typically more powerful than other summary-data-based MR approaches^19^. Furthermore, GSMR can account for linkage disequilibrium (LD) between SNPs^20^. We identified genetic instruments for each obesity-related trait from external genome-wide association studies (GWAS). Using these genetic instruments in conjunction with association statistics from a recent GWAS meta-analysis of meningioma^21,22^, we estimate the causal effect of each trait on meningioma risk.

## Results

We considered 11 obesity-related traits: BMI, basal metabolic rate (BMR), body fat percentage, diastolic blood pressure (DBP), fasting glucose, low-density lipoprotein cholesterol (LDL), high-density lipoprotein cholesterol (HDL), systolic blood pressure (SBP), total cholesterol, triglycerides and waist circumference. SNPs were excluded if there were data missing in either the two meningioma GWAS, or the data sets used to compute LD correlations (see Supplementary Table S1). We performed Heterogeneity in Dependent Instruments (HEIDI) outlier analysis^20^ to identify those genetic instruments violating MR pleiotropy assumptions, identifying three BMI, three BMR, two body fat percentage and two waist circumference SNPs, which were further excluded from the analyses (see Supplementary Table S1).

The number of SNPs used as IVs for each obesity-related trait, the proportion of variance explained (PVE) by these SNPs, and the mean and standard deviation (SD) for each obesity-related trait in the original discovery study are detailed in Table 1. The PVE was calculated using sample size estimates from each GWAS as well as effect sizes and standard errors for each SNP effect estimate. The SNPs used as IVs for each obesity-related trait, along with the association statistics for these SNPs in respect to disease risk are shown in Supplementary Table S2.

**Table 1:** Genetic instruments used to evaluate the causal relationship between obesity-related traits and meningioma risk.

Obesity-related trait	SNPs*	PVE (%)	Units	Source
BMI	927	7.5	kg/m^2^	Yengo *et al*.^43^
BMR	677	11.2	kilojoules	UK Biobank
Body fat percentage	434	5.8	%	UK Biobank
DBP	216	3.1	mmHg	UK Biobank
Fasting glucose	27	3.0	mmol/l	Scott *et al*.^42^
HDL	50	5.1	mg/dl	Willer *et al*.^44^
LDL	19	2.2	mg/dl	Willer *et al*.^44^
SBP	250	3.5	mmHg	UK Biobank
Total cholesterol	29	1.1	mg/dl	Willer *et al*.^44^
Triglycerides	19	1.9	mg/dl	Willer *et al*.^44^
Waist circumference	367	4.9	cm	UK Biobank

^*^After removal of SNPs during quality control and LD trimming.

One SNP reported in the original LDL discovery study (rs9411489) has been merged with another SNP (rs635634), and we therefore derived meningioma association statistics using rs635634.

**Figure 1:**
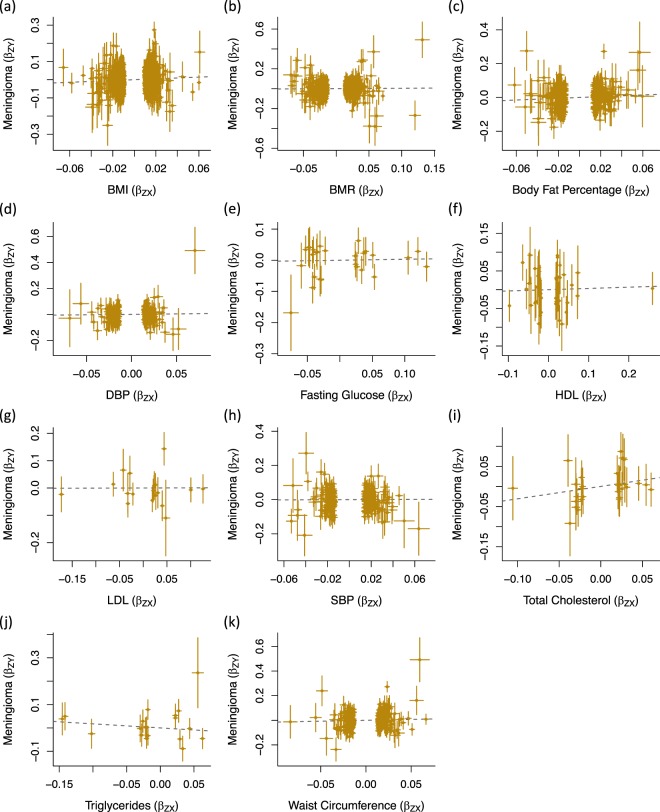
Effects of genetic instruments on obesity-related traits and meningioma risk. Shown are the results for (**a**) BMI, (**b**) BMR, (**c**) body fat percentage, (**d**) DBP, (**e**) fasting glucose, **(f**) HDL, (**g**) LDL, (**h**) SBP, (**i**) total cholesterol, (**j**) triglycerides and (**k**) waist circumference. For each SNP, the effect for the obesity-related trait is plotted against the effect for meningioma risk. Dashed lines represent GSMR estimates. Error bars represent one standard deviation. BMI: body mass index; BMR; basal metabolic rate; DBP: diastolic blood pressure; GSMR: Generalised Summary-data-based Mendelian Randomisation; HDL: high-density lipoprotein cholesterol; LDL: low-density lipoprotein cholesterol; SNP: single nucleotide polymorphism.

The association of each IV with the respective obesity-related trait and meningioma risk, and the log odds ratios (ORs) estimated by GSMR, are shown in Fig. 1. At a significance threshold of *P*<0.05, risk of meningioma was not associated with BMR (OR_SD_ = 1.04, 95% confidence interval [CI] = 0.87–1.24, *P* = 0.67), DBP (OR_SD_ = 1.07, 95% CI = 0.77–1.48, *P* = 0.68), fasting glucose (OR_SD_ = 1.03, 95% CI = 0.74–1.43, *P* = 0.85), HDL (OR_SD_ = 1.03, 95% CI = 0.81–1.33, *P* = 0.79), LDL (OR_SD_ = 1.00, 95% CI = 0.68–1.49, *P* = 0.98), SBP (OR_SD_ = 1.02, 95% CI = 0.75–1.39, *P* = 0.89), total cholesterol (OR_SD_ = 1.36, 95% CI = 0.79–2.34, *P* = 0.26), triglycerides (OR_SD_ = 0.84, 95% CI = 0.54–1.30, *P* = 0.44) or waist circumference (OR_SD_ = 1.16, 95% CI = 0.89–1.50, *P* = 0.27) (Table 2). There was however support for a positive relationship between both BMI (OR_SD_ = 1.27, 95% CI = 1.03–1.56, *P* = 0.028) and body fat percentage (OR_SD_ = 1.28, 95% CI = 1.01–1.63, *P* = 0.042) with meningioma risk, albeit non-significant after correction for multiple testing.

**Table 2:** GSMR results for each obesity-related trait and meningioma risk.

Obesity-related trait	OR (95% CI)	*P-*value
BMI	1.27 (1.03–1.56)	0.028
BMR	1.04 (0.87–1.24)	0.674
Body fat percentage	1.28 (1.01–1.63)	0.042
DBP	1.07 (0.77–1.48)	0.679
Fasting glucose	1.03 (0.74–1.43)	0.853
HDL	1.03 (0.81–1.33)	0.792
LDL	1.00 (0.68–1.49)	0.980
SBP	1.02 (0.75–1.39)	0.887
Total cholesterol	1.36 (0.79–2.34)	0.264
Triglycerides	0.84 (0.54–1.30)	0.436
Waist circumference	1.16 (0.89–1.50)	0.267

We used MR-Egger regression to assess whether directional pleiotropy could affect the causal estimates (see Supplementary Fig. S1 and Supplementary Table S3). In none of these analyses was the intercept of the causal effect significantly different from zero (*i.e. P* < 0.05), thereby providing no evidence that overall unbalanced pleiotropy biased results.

No obesity-related traits were identified by the MR-Egger as being associated with meningioma risk (see Supplementary Table S3). However, whilst a positive relationship between BMI and risk of meningioma was estimated by GSMR (OR_SD_ = 1.27, 95% CI = 1.03–1.56, *P* = 0.028), a non-significant negative relationship was estimated by MR-Egger regression (OR_SD_ = 0.93, 95% CI = 0.51–1.71, *P* = 0.83). As MR-Egger regression can be strongly influenced by a single outlying variant^23^, we conducted leave-one-out analysis to determine whether such a variant could explain this disparity (see Supplementary Table S4). When rs663129 was removed, the OR for BMI and risk of meningioma estimated by MR-Egger regression was 1.06 (95% CI = 0.57–2.00, *P* = 0.85). Conversely, the removal of rs663129 had little effect on the OR for BMI and risk of meningioma estimated by GSMR (OR = 1.29, 95% CI = 1.05–1.60, *P* = 0.017). This outlying variant therefore partly explains the difference in causal effects estimated by the two approaches. Single-SNP analyses of each obesity-related trait did not identify any other outlying SNPs (see Supplementary Fig. S2).

## Discussion

Twin and family studies have provided strong evidence for large genetic influences on obesity-related traits, with heritability estimates for BMI of between 50% and 90%, leaving the remaining variance attributable to environmental influences^24^. In our analysis, genetic markers correlated with 11 obesity-related traits were used as proxies for these traits.

As for the common cancers, there is increasing evidence that obesity increases the risk of developing rarer tumours, such as multiple myeloma^4,25^, inviting speculation that obesity and its related traits provide an environment supportive to tumorigenesis in general. As previously stated, observational epidemiological studies have provided mixed evidence on whether obesity influences the risk of meningioma development^5–11^. Diabetes and fasting serum glucose levels have been identified as being inversely related to meningioma risk in some studies^8,13,14^. Anti-diabetic treatment has however paradoxically been reported as being positively related to meningioma risk in one study^15^. Suggested mechanisms for the association of obesity and obesity-related traits with meningioma risk include chronic inflammation, and increased adipokine-mediated signalling, insulin signalling and insulin-like growth factor (IGF) signalling^26,27^. Obesity is associated with higher circulating levels of IGF-1, which is known to suppress apoptosis and stimulate tumour growth^28^. Higher expression of components of the IGF signalling system have also been observed in meningioma, suggesting a role for these proteins in the development of these tumours^29^. Furthermore, the transcription regulatory factors leptin and leptin receptor have been shown to be important in the proliferation and survival of patient-derived meningioma cell lines^30^. Leptin hormone concentrations are higher in individuals with high BMI, providing an alternative or complementary mechanism for the association of obesity with meningioma risk^30^. Although the long-term metabolic consequences of obesity-related traits are highly complex, these mechanisms are compatible with the generic effect of obesity on cancer risk.

Oestrogen pathways also provide a biological explanation for the relationship between obesity and some cancers, including breast and endometrial^31^. Oestrogen interacts with IGF in the brain^32^ and such hormonal factors may account for the two-fold increase in meningioma risk observed in women when compared to men^33^. Both hormone replacement therapy^34–37^ and oral contraceptive use^35–37^ have however been inconsistently associated with meningioma risk. In breast cancer, a higher lifetime exposure to oestrogen, as measured using age at menarche and menopause, is associated with increased disease risk^38^. Reported associations between lifetime oestrogen exposure and meningioma risk have however not been consistent^3,39^, suggesting a complex relationship between hormone exposure, obesity and risk of meningioma.

Our MR analysis provides evidence for a causal relationship between obesity, specifically higher BMI and body fat percentage, and meningioma risk. An advantage of using MR to identify causal relationships between environmental factors and disease risk is that the random allocation of genetic variants at conception avoids reverse causation, which may have biased causal estimates in previous observational epidemiological studies. MR-Egger analysis of each obesity-related trait did not identify bias caused by directional pleiotropy (see Supplementary Table S3). Therefore, whilst we cannot exclude the possibility that confounders such as type-2 diabetes medication use may be having some effect on the causal estimates, it is unlikely that they are a substantial source of bias. Some potential weak instrument bias is quantified by the low (<10) first-stage F-statistics for BMI, BMR, body fat percentage, DBP, SBP, total cholesterol and waist circumference, with F-statistics of 0.9, 2.0, 1.6, 1.7, 1.6, 4.6 and 1.6 respectively. However, F-statistics for fasting glucose, LDL, HDL and triglycerides indicates that these are strong IVs, with respective values of 13.0, 13.7, 12.3, 11.2, and are hence less likely to be affected by weak instrument bias^40^.

Our study does however have limited power to detect causal effects for some of the obesity-related traits (Table 3). In particular, our study had less than 80% power to identify ORs of 0.67 or 1.50 for DBP, fasting glucose and total cholesterol. Thus, interpretation of the null results found for these traits in this study are limited, as modest associations between these traits and meningioma risk cannot be excluded.

**Table 3:** Odds ratios required for 80% study power.

Obesity-related trait	OR low	OR high
BMI	0.76	1.32
BMR	0.80	1.25
Body fat percentage	0.73	1.37
DBP	0.65	1.53
Fasting glucose	0.65	1.54
HDL	0.78	1.28
LDL	0.70	1.43
SBP	0.67	1.49
Total cholesterol	0.62	1.61
Triglycerides	0.69	1.45
Waist circumference	0.71	1.40

In conclusion, our analysis provides further evidence that higher BMI and body fat percentage are risk factors for meningioma.

## Methods

All data used in this study were retrieved from publicly available summary statistics of published GWAS and we therefore did not seek ethical approval for this specific project. This study used no individual-level data.

### Genetic instruments for obesity-related traits

We identified genetic instruments for each obesity-related trait suitable for use in MR analyses by considering the largest GWAS or meta-analysis of each trait conducted to date. Only continuous obesity-related traits were considered, as MR analysis of a binary exposure with a binary outcome using summary data in a two-sample setting can result in inflated causal estimates^41^. We identified a panel of SNPs associated with each trait at a genome-wide level of significance (*P* < 5 × 10^–8^) and minor allele frequency >0.01. Only studies of individuals of European ancestry were considered. Genetic instruments for fasting glucose were retrieved from an analysis by the Meta-Analysis of Glucose and Insulin related traits Consortium (MAGIC)^42^, instruments for BMI from the Genetic Investigation of Anthropometric Traits (GIANT) consortium^43^, instruments for HDL, LDL, total cholesterol and triglycerides from the Global Lipids Genetic Consortium (GLGC)^44^, and instruments for BMR, body fat percentage, DBP, SBP and waist circumference were obtained from GWAS using data from UK Biobank^45–47^. For each SNP used as a genetic instrument, we obtained the estimated per-allele effect on the obesity-related trait, the standard error of this estimate, and the chromosomal position of the SNP (see Supplementary Table S2). In order to mitigate against co-linearity between SNPs, which can bias causal estimates, we used MR-Base to exclude correlated SNPs at a threshold of r^2^ > 0.01, retaining the SNPs with the strongest effect on the respective obesity-related trait^46,48^. Effect size estimates and the standard errors of these estimates were standardized to represent the per-allele effect of the SNP on the exposure in standard deviation units^20^.

### Meningioma association statistics

We examined the association of the respective genetic instruments for each obesity-related trait with risk of meningioma using data from a recent meta-analysis of meningioma GWAS. This GWAS meta-analysis includes 1,606 meningioma patients and 9,823 controls of European descent from two independent GWAS datasets (see Supplementary Table S5)^22^, and after imputation comprises >9 million genetic variants. Details of the two independent GWAS datasets, including participant recruitment and the quality control procedures conducted, have been reported previously^22^. We used MR-Base to ensure that the effect of each SNP on the obesity-related trait and meningioma risk corresponded to the same allele^46^.

### Evaluating causal relationships using Mendelian randomisation

For each obesity-related trait, we used GSMR to estimate the OR of meningioma per unit of SD increment of each considered risk factor^20^. The GSMR R-package contains an implementation of the HEIDI outlier test, which can be used to identify loci that influence multiple phenotypes (*i.e*. the exposure and outcome considered in an MR analysis)^20^. LD correlations between SNPs were estimated using data from the 1000 Genomes Project^49^ and the UK10K^50^. We used HEIDI outlier tests to identify and exclude SNPs that may have pleiotropic effects on each obesity-related trait and meningioma risk (see Supplementary Table S1).

To derive conclusions concerning which obesity-related traits were causally related to meningioma risk, we considered *P* < 0.05 as a global significance level. To correct for multiple testing we applied Bonferroni-correction imposing a significance threshold of 0.0045 (*i.e*. 0.05/11 traits). The power of any MR analysis depends on the PVE captured by the SNPs used as IVs and we therefore estimated the power of each analysis using the method from Burgess *et al*.^51^. All statistical analyses were conducted using R (v3.1.2).

We used MR-Egger regression to further assess the violation of MR assumptions caused by directional pleiotropy (see Supplementary Fig. S1 and Supplementary Table S3)^52^. Significant difference between the slope estimated by MR-Egger regression and the intercept was considered evidence of violation. SNPs failing the HEIDI outlier tests were not included in the MR-Egger regression analyses. Furthermore, we conducted single-SNP MR-Egger analyses and display the results in funnel plots to allow the visual identification of any outlying SNPs (see Supplementary Fig. S2)^52^. We used leave-one-out analysis, as per Burgess and Thompson^23^, to assess whether outlying variants could be influencing causal estimates from MR-Egger regression.

## Electronic supplementary material

Supplementary Figures 1-2

Supplementary Tables 1-5

## Data Availability

Summary statistics for the obesity-related trait GWAS using UK Biobank data are available from http://www.nealelab.is/uk-biobank/. Genotype data from The Resource for Genetic Epidemiology Research on Aging can be downloaded from The Database of Genotypes and Phenotypes (dbGaP) (accession code phs000674.v2.p2). The 1000 Genomes Project and UK10K imputation panel data are available from the European Genome-phenome Archive (EGA, accession EGAD00001000776). Remaining data are available upon request.
