# Supplementary material for: Mendelian randomization provides support for obesity as a risk factor for meningioma
Source: Sci Rep. 2019 Jan 22;9:309. doi: 10.1038/s41598-018-36186-6 (PMC6343031; doi:10.1038/s41598-018-36186-6)
Supplement: Supplementary file 1 — Supplementary Figures 1-2 [file 41598_2018_36186_MOESM1_ESM.pdf]

SUPPLEMENTARY FIGURES

Mendelian randomization provides support for obesity as a risk factor for meningioma

Hannah Takahashi, Alex J. Cornish, Amit Sud, Philip J. Law, Linden Disney-Hogg, Lisa Calvocoressi, Lingeng Lu, Helen M. Hansen, Ivan Smirnov, Kyle M. Walsh, Johannes Schramm, Per Hoffmann, Markus M. Nöthen, Karl-Heinz Jöckel, Joellen M. Schildkraut, Matthias Simon, Melissa Bondy, Margaret Wrensch, Joseph L. Wiemels, Elizabeth B. Claus, Clare Turnbull, Richard S. Houlston.

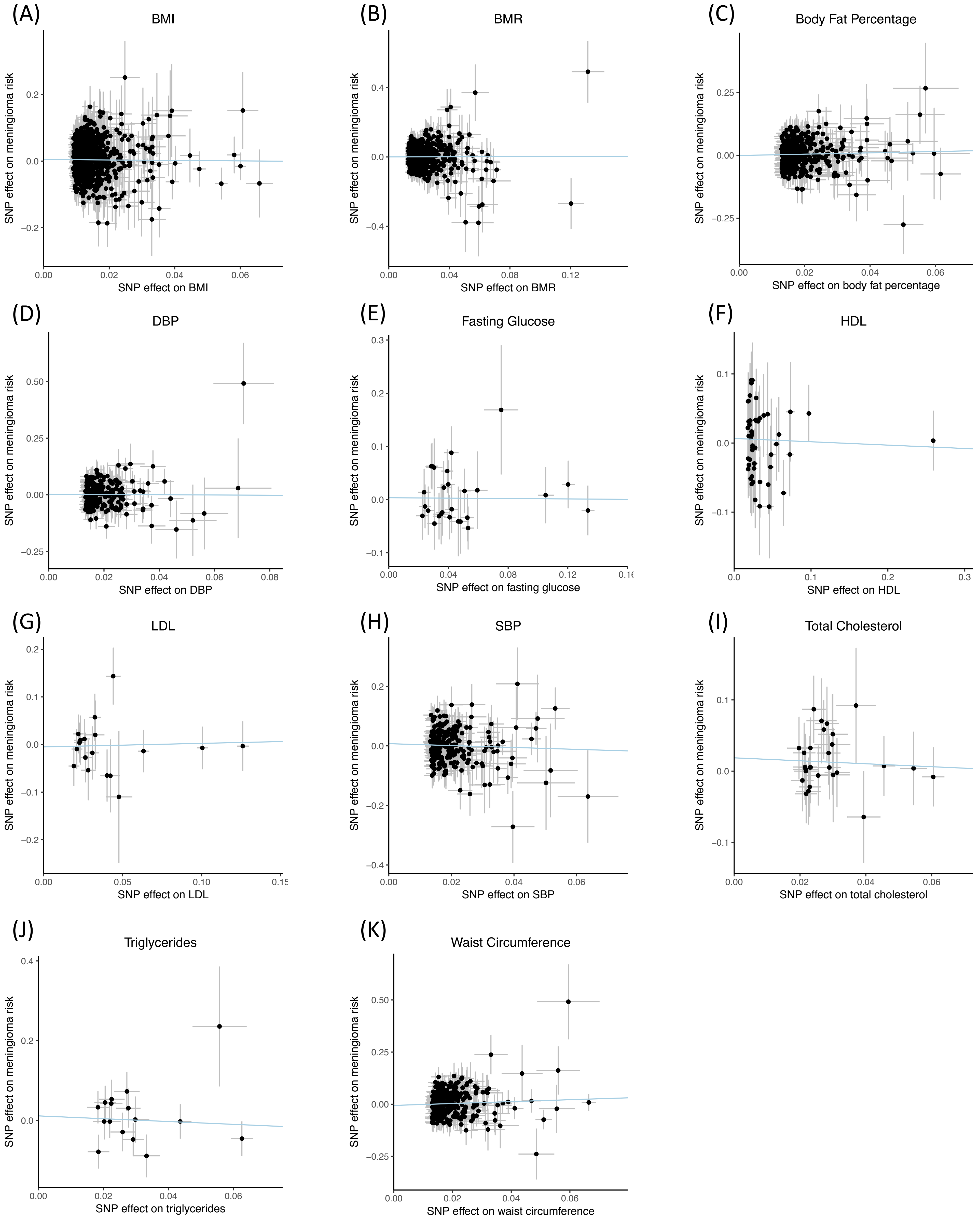

Supplementary Figure S1: Scatter plots of genetic associations with the obesity-related traits against genetic associations with meningioma risk. Shown are the results for (A) BMI, (B) BMR, (C) body fat percentage, (D) DBP, (E) fasting glucose, (F) HDL, (G) LDL, (H) SBP, (I) total cholesterol, (J) triglycerides and (K) waist circumference. Blue line represents MR-Egger estimate.

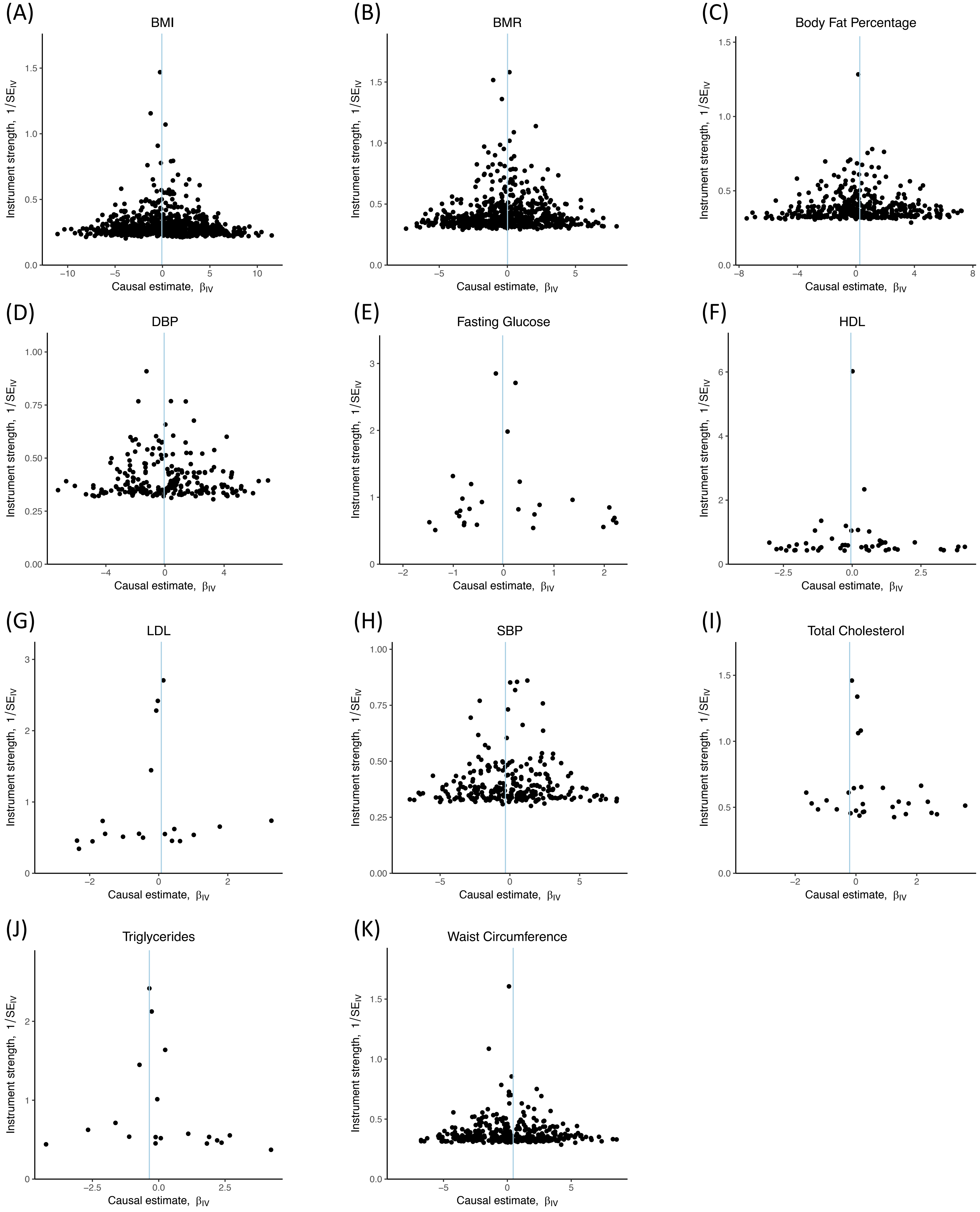

Supplementary Figure S2: **Funnel plots showing for each SNP the causal estimate of the obesity-related trait on meningioma, and the strength of the instrument.** Shown are the results for (A) BMI, (B) BMR, (C) body fat percentage, (D) DBP, (E) fasting glucose, (F) HDL, (G) LDL, (H) SBP, (I) total cholesterol, (J) triglycerides and (K) waist circumference. Blue line represents causal estimate from MR-Egger analysis when all SNPs in plot are considered.
